# Comorbidities and Age Are Associated With Persistent COVID-19 PCR Positivity

**DOI:** 10.3389/fcimb.2021.650753

**Published:** 2021-04-06

**Authors:** Mohammed Aldhaeefi, Zabreen Tahir, David J. Cote, Saef Izzy, Joseph El Khoury

**Affiliations:** ^1^ Department of Pharmacy Services, Brigham and Women’s Hospital, Boston, MA, United States; ^2^ Department of Neurology, Neurocritical Care, Brigham and Women’s Hospital, Boston, MA, United States; ^3^ Center for Immunology & Inflammatory Diseases, Massachusetts General Hospital, Boston, MA, United States; ^4^ Harvard Medical School, Boston, MA, United States; ^5^ Department of Medicine, Division of Infectious Diseases, Massachusetts General Hospital, Boston, MA, United States

**Keywords:** comorbidities, age, COVID19, positivity, PCR

## Abstract

**Objectives:**

The impact of demographics and comorbidities on the duration of COVID-19 nasopharyngeal swab PCR positivity remains unclear. The objective of our analysis is to determine the impact of age, intensive care unit (ICU) admission, comorbidities, and ethnicity on the duration of COVID-19 PCR positivity among hospitalized patients in a large group of hospital.

**Method:**

We studied 530 patients from a large hospital system and time to SARS-CoV-2 virus RNA PCR negativity at any-time during hospitalization or following discharge from the hospital was the primary endpoint. We included patients 18 years or older who tested positive for COVID-19 during an inpatient, outpatient, or emergency room visit between February 1, 2020, and April 14, 2020.

**Results:**

Overall, 315 (59.4%) of our patient population continued to have a positive SARS-CoV-2 virus RNA PCR 4 weeks after the initial positive test. We found that age>70 years, chronic kidney disease, hypertension, hyperlipidemia, obesity, or coronary artery disease are associated with persistent PCR positivity for more than 4 weeks after initial diagnosis.

**Conclusion:**

Age, and the presence of co-morbidities should be taken into consideration when interpreting a positive COVID PCR test.

## Introduction

Coronavirus Disease 2019 (COVID-19) was declared a public health emergency by the World Health Organization (WHO) in January 2020 (Guan et al., 2020). Patients with COVID-19 present with a wide variety of symptoms, such as cough, shortness of breath, headache or body aches, fatigue, and loss of taste or smell (Guan et al., 2020; Izzy et al., 2020; Roberts et al., 2020). As of December 2020, COVID-19 resulted with over 67.8 million confirmed cases worldwide in 215 countries with over 1.5 million deaths (Worldometers.info., 2020).

Few studies were conducted to determine the duration of COVID-19 PCR positivity following initial diagnosis (Million et al., 2020; Scola, 2020; Van Kampen et al., 2020; Zhou, 2020). However, there are inconsistencies in the findings among these studies. SARS-CoV-2 virus RNA was detectable in severe infections up to 20 days post symptoms (Van Kampen et al., 2020) but could be undetectable as early as 3-8 days post symptoms in mild to moderate infections (Scola, 2020). More extended periods were reported with immunocompetent patients with time to negative PCR results of 15-37 days post-onset of treatment (Zhou, 2020; Million et al., 2020). The impact of age, intensive care unit (ICU) admission, comorbidities, and ethnicity remain unclear.

The objective of our analysis is to determine the impact of age, intensive care unit (ICU) admission, comorbidities, and ethnicity on the duration of COVID-19 PCR positivity among hospitalized patients in a large group of hospital. 

## Method

### Data Source

Patient information was retrieved retrospectively using an electronic health record (Epic Systems, Verona, WI) shared by all Mass General Brigham healthcare system institutions. Mass General Brigham is a not-for-profit healthcare system affiliated with Harvard Medical School that comprises 12 hospitals across eastern Massachusetts. We included patients 18 years or older who tested positive for COVID-19 during an inpatient, outpatient, or emergency room visit between February 1, 2020, and April 14, 2020. Patients were diagnosed as infected with COVID-19 if SARS-CoV-2 RNA was detected in upper or lower respiratory specimens by nucleic acid testing (NAT) assays designated for emergency use authorization (EUA) by the Food and Drug Administration (FDA) and in accordance with the Centers for Disease Control and Prevention (CDC) guidelines. Each assay targets at least one SARS-CoV-2 gene region; positive results are reported for each assay as defined by the manufacturer or reference laboratory. The repeated tests reported were done using the same real time quantitative PCR platform. Cycle threshold (Ct) values were included when the results were available. A Ct of 41 or more was considered negative. This study was approved by the Mass General Brigham Institutional Review Board (Protocol: 2020P001071). 

### Endpoint

Time to SARS-CoV-2 virus RNA PCR negativity at any-time during hospitalization or following discharge from the hospital was the primary endpoint. We extracted multiple covariates and examined their effect on the duration of SARS-CoV-2 virus PCR positivity. The following covariates were obtained from the electronic health records for all patients: age, race/ethnicity (White, African American, Hispanic, Asian or others), smoking status, ICU admission status, obesity (as measured by body mass index [BMI] of > 30 kg/m^2^), and the presence of recorded comorbidities including diabetes mellitus, hyperlipidemia, coronary artery disease, obstructive lung disease, interstitial lung disease, congestive heart failure, chronic kidney disease, autoimmune diseases, transplant, cerebrovascular disease, and cancer.

### Statistical Analysis

Descriptive analyses of variables are presented as proportions or medians with interquartile range (IQR) for all endpoints. We used the analysis of covariance (ANCOVA) to determine the effect of multiple covariates on the duration of SARS-CoV-2 virus PCR positivity. Logistic regression was used to identify the predictors of time to negative results (<=4 weeks versus >4 weeks post 1^st^ positive result). We chose 4 weeks as a cutoff point since 40.6% of our patient population reversed to negative by week 4. Kaplan-Meier plots were used to describe the time to a negative test result with a log-rank test to compare the difference between the presence of different comorbidities and demographics. Statistical significance was defined as p<0.05 for all analyses, and all statistical analysis was completed using R v 3.6.1.24.

## Results

Of 1490 patients who had an initial positive PCR test for COVID-10, 530 (36%) patients were retested and had documented COVID-19 negative results and were included in the analysis. Overall, 291 (54.9%) of these patients were male, with a median age of 61 (IQR: 49-73) years. Among our study population 41.3% were white 32.3% were Latinx patients and 15.1% were African American patients. The median length of inpatient stay was 12 (IQR: 6-19) days, and 236 (44.5%) of our patients were admitted to the ICU with a median duration of ICU stay of 10 (IQR: 5-15) days. The median BMI of our patient population was 29.2 (IQR: 25.5-33.9). The most commonly reported comorbidities were hypertension (51.5%), hyperlipidemia (38.5%), diabetes (36.8%), and obesity (20.2%). Patients comorbidities are summarized in **Table 1**.

**Table 1:** Patients Comorbidities.

Comorbidity^*^	Number of Patients (n=530)
Hypertension	273 (51.5)
Hyperlipemia	204 (38.5)
Diabetes	195 (36.8)
Obesity	107 (20.2)
Obstructive lung disease	79 (14.9)
Coronary artery disease	56 (10.6)
Chronic kidney disease	56 (10.6)
Obstructive sleep apnea	34 (6.4)
Congestive heart failure	31 (5.8)
Autoimmune disease	25 (4.7)
Cerebrovascular disease	24 (4.5)
Transplant	15 (2.8)
Interstitial lung disease	1 (0.2)

*n (%).

**Figure 1:**
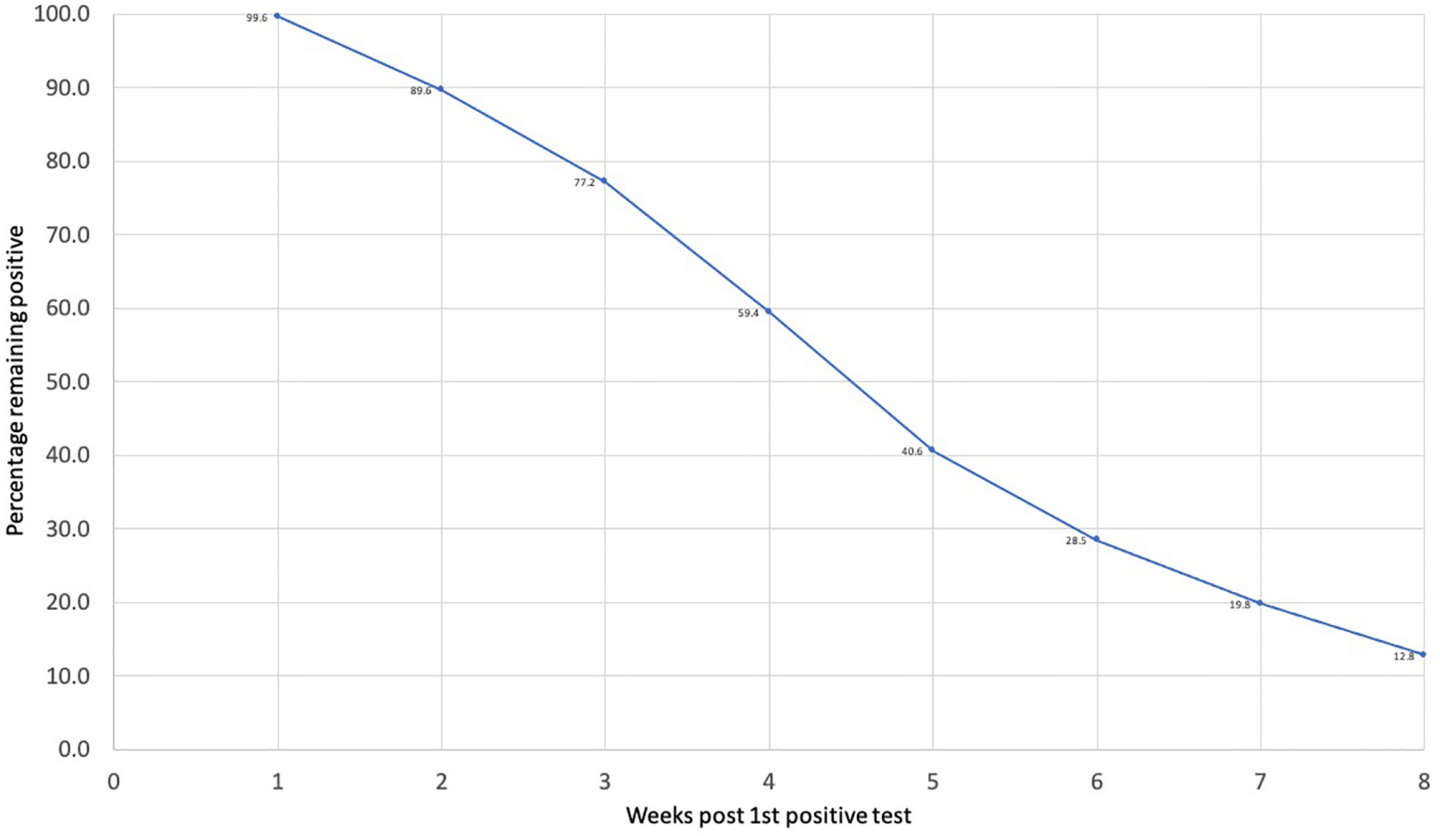
Percent of patients remaining positive up to 8 weeks after the first positive PCR test.

Overall, 315 (59.4%) of our patient population continued to have a positive SARS-CoV-2 virus RNA PCR 4 weeks after the initial positive test (**Figure 1**). Only 215 (40.6%) patients reverted to negative by week 4. Median age of those who reverted was 57 (IQR: 45-70) years. Among these patients, 81 (37.7%) were white patients and 65 (30.2%) Latinx patients and 38 (17.7%) African American. The PCR test continued to be positive after 8 weeks of the initial positive test in 68 (12.8%) patients (**Figure 1**). These patients had a median age of 64 (IQR: 53.8-76) years, and 44% of them were White patients, and 36.8% were Latinx patients and 14.7% African American.

Kaplan-Meier analysis showed that race, age, presence of hypertension, hyperlipidemia, obstructive lung disease, coronary artery disease, and chronic kidney disease were associated with time to conversion SARS-CoV-2 virus RNA PCR negative results (p<0.05 for all).

Univariate logistic regression analysis (**Table 2**) showed that age >70 years (OR: 1.9, 95% CI 1.1-3.3, p-value=0.01), presence of hypertension (OR: 1.4, 95% CI 1.0-2.1, p-value=0.03), hyperlipidemia (OR: 1.5, 95% CI 1.0-2.1, p-value=0.04), obesity (OR: 1.6, 95% CI 1.0-2.5, p-value=0.04), coronary artery disease (OR: 2.2, 95% CI 1.2-4.3, p-value=0.01), and chronic kidney disease (OR: 2.7, 95% CI 1.4-5.4, p-value=0.003) were independently associated with higher probability of having a positive COVID-19 results after four weeks from the 1^st^ positive result. No significant association was shown with gender, race, smoking status or ICU admission.

**Table 2:** Predictors of negative test after 4 weeks compared to those within 4 weeks.

	Univariable	p-value
	OR (95% CI)	
**Gender**		
Female	Reference	
Male	1.2 (0.9-1.8)	0.21
**Age category**		
18-40	Reference	
41-70	1.4 (0.8-2.2)	0.23
>70	1.9 (1.1-3.3)	**0.01**
**Race/ethnicity**		
White	Reference	
Hispanic	0.97 ().7-1.5)	0.94
Black	0.62 (0.4-1.0)	0.07
Other	0.54 (0.2-1.3)	0.15
Unknown	0.52 (0.3-1.1)	0.07
**Smoking status**		
Former	1.7 (0.7-3.9)	0.22
Current	Reference	
Never	1.5 (0.7-3.4)	0.33
**ICU admission**		
No	Reference	
Yes	1.24 (0.9-1.8)	0.23
**Medical comorbidities**		
Hypertension	1.4 (1.0-2.1)	**0.03**
Hyperlipidemia	1.5 (1.0-2.1)	**0.04**
Diabetes mellitus	1.4 (0.9-2.0)	0.09
Obesity	1.6 (1.0-2.5)	**0.04**
Obstructive lung disease	1.3 (0.8-2.2)	0.29
Coronary Artery disease	2.2 (1.2-4.3)	**0.01**
Cerebrovascular disease	0.9 (0.4-2.3)	0.91
Chronic kidney disease	2.7 (1.4-5.4)	**0.003**
Congestive heart failure	1.3 (0.6-2.8)	0.55
Obstructive sleep apnea	1.5 (0.7-3.2)	0.32
Auto-immune disease	2.7 (1.1-8.2)	0.05
Cancer	1.3 (0.7-2.5)	0.51
Transplant	0.8 (0.3-2.2)	0.63

Bolded values are statistically significant.

**Figure 2:**
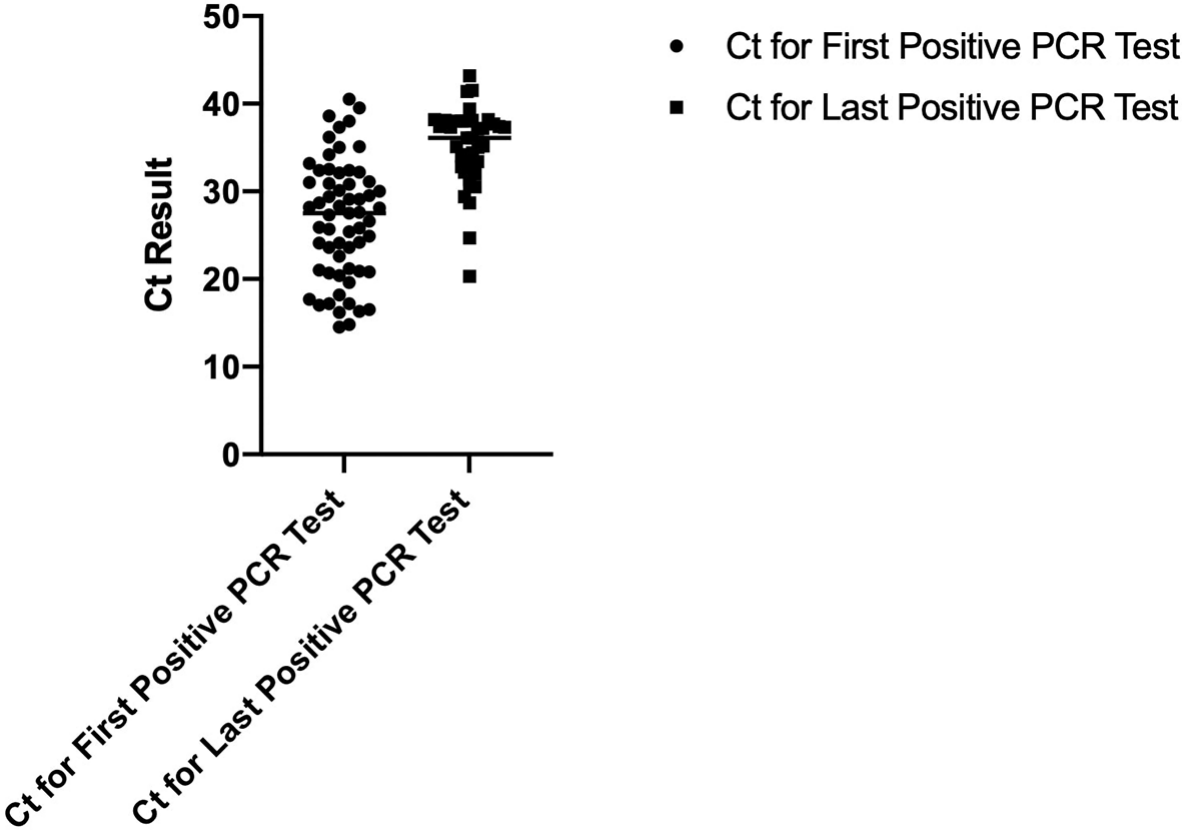
Dot-plot of mean SARS-CoV-2 Ct values for the first and last positive PCR tests.

To determine if a persistent positive PCR test is associated with persistently elevated viral RNA, we evaluated the cycle threshold (Ct) values for the first and last positive PCR tests for 71 patients. **Figure 2** displays the dot-plot of the mean SAR-CoV-2 Ct values for the first and last positive PCR tests. The mean Ct value for the first and last positive PCR tests were 26.63 and 35, respectively (p-value=<0.0001) indicating that there was a significant reduction in viral RNA levels even though the test remained positive. 

## Discussion

In this study, we found that age >70 years, presence of hypertension, hyperlipidemia, obesity, coronary artery disease, or chronic kidney were associated with an increased likelihood of having a longer time to negative COVID-19 test result after initial positive result.

The effect of age on time to first SARS-CoV-2 virus RNA PCR negative result was reported in previous studies (Guan et al., 2020; Voinsky et al., 2020). Voinsky et al. demonstrated that patients older than 30 years old require significantly longer periods to have the first negative PCR result in comparison to those younger than 30 years old (Voinsky et al., 2020). Additionally, Hu et al. showed the same pattern among patients who were >45 years old (Guan et al., 2020). Our findings extend these studies and suggest that age of >70 years old could potentially impact the period of the time to first negative PCR result and reduced the probability of having negative PCR results within 4 weeks.

It is not clear if patients with persistently positive PCR test have persistent live virus or the positive test reflects residual viral RNA from dead virus. Patients with delayed SARS-CoV-2 virus RNA clearance have demonstrated a higher mortality rate in comparison to those with an early clearance (Zhou, 2020). Delayed SARS-CoV-2 virus RNA clearance was found among elderly patients and patients with coronary artery disease or hypertension (Chen et al., 2020; Fu et al., 2020). Interestingly, in one study with ICU patients, non-survivors experienced delayed clearance, and 75.9% of those patients were >60 years old with multiple comorbidities, although no statistical analysis was done (Wang et al., 2020). Our data support these findings and extend them to non ICU patients. In some studies, diabetes and cancer delayed the clearance; however, we did not find any statistical difference among these groups in our analysis (Chen et al., 2020; Tian et al., 2020).

Based on our analysis, the last positive PCR test had statistically significant lower Ct values in comparison to the first positive PCR test. This suggests a lower viral load for the last positive PCR test. The evidence supporting the correlation between Ct values and patient’s infectivity is conflicting. However, two studies concluded that lower Ct values were associated with higher probability of a positive viral culture and infectivity (Bullard et al., 2020; Van Kampen et al., 2020). La Scola et al. showed patients with Ct <33-34 are infective, however; Bullard et al. demonstrated patients with Ct <24 could be still contagious (Bullard et al., 2020; Van Kampen et al., 2020). Our patients were likely contagious during their first positive PCR test based on mean Ct value of 26.63, but were potentially non-contagious when they had their last positive PCR test based on mean Ct value of 35. Nonetheless, because of the limited studies regarding the correlation between infectivity and Ct values, we favor caution when interpreting such values.

Our study has some possible limitations. First our study may underrepresent outpatient COVID-19 patients who do not seek medical attention or whose medical data are stored at other facilities. Also, not all of our patients who tested positive initially were retested. Lastly, Ct values were not documented for all patients. Strengths of our study include a large, diverse study population of COVID-19-positive patients from a wide geographic region that allowed us to analyze a large number of Latinx, African American, and White patients. We were also able to collect detailed sets of variables on each patient, which allows for multivariable adjustment. 

## Conclusion

Our findings suggest that patients >70 years of age and those with chronic kidney disease, hypertension, hyperlipidemia, obesity, or coronary artery disease have persistently positive PCR tests suggesting delayed SARS-CoV-2 virus RNA clearance. Age, and the presence of co-morbidities should be taken into consideration when interpreting a positive COVID PCR test. Persistently positive tests correlated with higher PCR Ct values suggesting decreased viral RNA. Additional studies on whether such persistent positive tests reflect persistent viral presence vs. residual viral material from dead virus need to be done.

## Data Availability Statement

The raw data supporting the conclusions of this article will be made available by the authors, without undue reservation.

## Author Contributions

All authors have contributed to the data collection, analysis and manuscript writing. All authors contributed to the article and approved the submitted version.

## Conflict of Interest

The authors declare that the research was conducted in the absence of any commercial or financial relationships that could be construed as a potential conflict of interest.
